# Application of three dimensional (3D) curved multi‐planar reconstruction images in 3D printing mold assisted eyebrow arch keyhole microsurgery

**DOI:** 10.1002/brb3.1785

**Published:** 2020-08-14

**Authors:** Sheng‐Jun Li, Fang Wang, Wei Chen, Ying Su

**Affiliations:** ^1^ Department of Neurosurgery Linyi Central Hospital Linyi China; ^2^ Department of Emergency Linyi Central Hospital Linyi China

**Keywords:** 3D printing, aneurysm, eyebrow arch keyhole approach, minimally invasive surgery, Three‐dimensional surface multi‐planar reconstruction

## Abstract

**Objective:**

The application of multi‐planar reconstruction of three dimensional (3D) curved surface in microsurgery of 3D printing mold assisted eyebrow arch keyhole approach was studied.

**Methods:**

Eighty patients with intracranial aneurysms who underwent treatment at our hospital were enrolled. The patients were divided into two groups: the traditional eyebrow keyhole approach microsurgery group (38 cases in the conventional treatment group) and the three‐dimensional curved surface multi‐plane reconstruction image combined with 3D printing technology assisted eyebrow keyhole approach microsurgery group (42 cases in the 3D printing assisted treatment group). The Hunt‐Hess classification was used to make a preliminary estimation of the patient's condition. The 3D curved multi‐planar reconstruction method was used to assist the surgical plan; CT scan was used to establish a 3D printing mold, and the patient's condition and surgical plan were accurately analyzed before surgery. The operative time and the size of the incision area were recorded; postoperative GOS score and postoperative complications were statistically investigated.

**Results:**

The 3D printing assisted treatment group (70.13 ± 15.56), (411.26 ± 10.38) mm^2^, the operative time and incision area were significantly shorter than the conventional treatment group (120.35 ± 20.46), (663.55 ± 13.54) mm^2^, *p* < .05); the GOS score showed that the 3D printing‐assisted treatment group was significantly higher than the conventional treatment group (*p* < .05). The postoperative complication rate was significantly lower in the 3D print‐assisted treatment group (9.52%) than in the conventional treatment group (47.36%, *p* < .05); the cure of intracranial aneurysms in the 3D printing assisted treatment group was more thorough than that in the conventional treatment group, and the difference was significant (*p* < .05).

**Conclusion:**

Compared with the conventional eyebrow arch‐hole approach microsurgery, the 3D surface multi‐planar reconstruction image combined 3D printing assisted technology was safer and more effective, and the postoperative recovery was better and the incidence of complications was lower.

## Graphical abstract

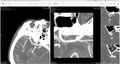

## 1 INTRODUCTION

A brain aneurysm is a representative cerebrovascular disease (Rehder & Cohen, 2017). From the perspective of the history of cerebral aneurysm treatment, surgical treatment has improved since the ligation of the common carotid artery to treat ipsilateral cerebral aneurysms (Li & Shi, 2012). The era of endovascular treatment began with detachable coils, and endovascular intervention is considered the primary treatment of cerebral aneurysm, which has yielded a lower complication rate and satisfactory outcomes compared with surgical clipping (Lan, Zhang, & Zhu, 2017; Yu, Yao, Zheng, & Kang, 2015). The keyhole approach has recently emerged, and Donald Wilson and Mario Brock first attempted a limited craniotomy as a preliminary step in the 1970s, and John Jane described an improvement to the sacral approach. Multiplanar reconstruction is a method for displaying 3 dimensional (3D) datasets and allows for the generation of cross‐sectional images such as the original two‐dimensional coronal, sagittal, and oblique images (Hong, Cheng, Wang, & Feng, 2017; Hwang, Park, & Park, 2017). Multiplanar reconstruction image of a curved surface is perpendicular to the sectional image of a specific curve made by the user (Abosch, Tyrrell, & Lamborn, 1998). We used a 3D surface multiplanar reconstruction technique to create a cross‐sectional image perpendicular to the sylvian surface along the sphenoid ridge. Preoperative 3D modeling can diagnose and simulate surgery more effectively. In recent years, advances in 3D reconstruction techniques in radiology have provided tools for virtual surgical planning. Rapid prototyping is the precise replication of virtual models obtained by computed tomography (CT), achieving submillimeter accuracy (Kurucz, Opitz, Buchfelder, & Ganslandt, 2018; Orman, Wagner, & Seeburg, 2015). In this study, we used a 3D curved multi‐planar reconstruction image combined with 3D printing technology to assist the eyebrow arch keyhole microsurgery in the treatment of intracranial aneurysms and evaluated its feasibility, effectiveness, and safety in the surgical plan.

## 2 PATIENTS AND METHODS

### 2.1 Patients

This clinical trial included 80 patients with intracranial aneurysms who were treated at our hospital between November 2017 and January 2019. They included 42 males and 38 females with a mean age of 41.43 ± 8.43 years. Thirty cases had anterior communicating aneurysm, 22 cases had intracranial bifurcation aneurysm, 22 cases had internal carotid artery post‐tracheal aneurysm, and 6 cases had middle cerebral artery aneurysm. Most patients lacked a family history of genetic disease and had normal body movements, and no serious heart disease and mental illness. It was guaranteed to complete the experimental study. The inclusion criteria were age 25 to 60 years; male or female; intracranial aneurysm patients and no previous surgical treatment; aneurysm had been formed; aneurysm number 1 ~ 5; follow‐up time of more than 12 months. Patients were excluded if they have the following diseases: history of severe brain disease; history of brain surgery; history of facial plastic surgery; patients with severe heart disease; patients with mental illness; patients with epilepsy or Parkinson's syndrome. All subjects and their families signed informed consent before surgery; the study was approved by the ethics committee; the patient's medical records were kept confidential to protect the privacy of the subjects.

Eight patients were divided into two groups, there were no significant differences in age, sex, and body weight between the two groups. 38 patients in the conventional treatment group underwent traditional eyebrow keyhole approach microsurgery, and 42 patients in the 3D printing assisted treatment group received three‐dimensional curved multiplanar reconstruction images combined with 3D printing technology to assist eyebrow keyhole approach microsurgery.

### 2.2 Preoperative Hunt‐hess classification of patients

In order to compare the postoperative status and preoperative planning of the two groups, we performed Hunt‐Hess classification during the hospitalization, 7 cases with grade 0, 55 cases with grade 1–3, and 18 cases with grade 4–5. There were 54 patients with 2 aneurysms, 15 patients with 3 aneurysms, 9 patients with 4 aneurysms, and 2 patients with 5 aneurysms and above.

### 2.3 Generating a 3D surface multiplanar reconstructed image

**FIGURE 1:**
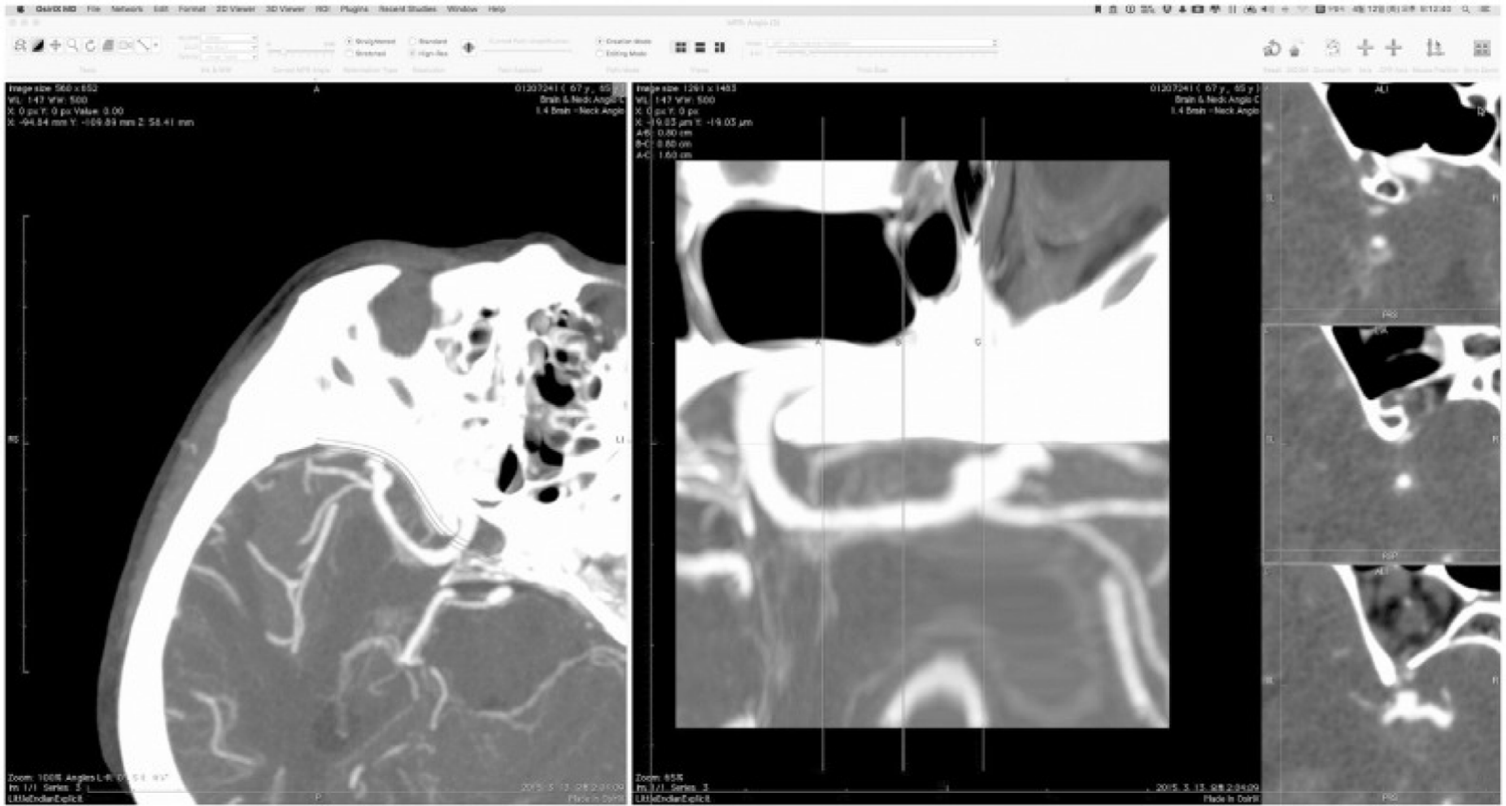
3D multi‐planar reconstruction image

An axial image (1.5 mm thickness) from brain CT angiography was output in DICOM format and imported into OsiriX MDTM imaging software (Pixmeo Inc., Geneva, Switzerland). The first step was to create a 3D multiplanar reconstructed image by clicking the 3D Surface Multiplanar Reconstruction button. The second step was to define a curve along the sphenoid ridge on the 3D multiplanar reconstructed image. The final step involved adjusting the settings of the work screen by changing several factors. Then, we observed the continuous multi‐planar reconstruction image perpendicular to the curve by dragging the position bar on the work screen. The left panel shows the right MCA aneurysm on a 3D multiplanar reconstructed image. The middle panel shows a straightened image of the curve on the sphenoid ridge, and the right three panels show an image perpendicular to the straight line at three points (Figure 1). In addition, the entire course and the depth change of the MCA in the SF were visualized. These images are perpendicular to the sylvian surface and have the same left‐right orientation as the surgeon's view.

### 2.4 3D model printing

A CT scan of patients with intracranial aneurysms was performed. Raw CT data were stored in DICOM format and reconstructed using Mimics software v17.0, and positioned by adjusting the threshold to show the integrity of the surrounding skull and bone structure. The Region Growing command was used to separate bones and soft tissues and build a skull model. The pixel set of the skull was processed using a computed 3D Form Mask command to produce a contralateral mirror image that served as a 3D model of the intracranial aneurysm side. Create a Mask set for each fragment was created, and the 3D object was calculated by Mask. The 3D model of the patient's head was calculated using Unite Boolean, and then the design data were imported into the 3D printing software (Cura Software v15.02) in STL format. After forming the 3D digital model, we saved it in Gcode format and exported it to a 3D printer (3D ORTHO Waston Med Inc., Changzhou, Jiangsu, China). Finally, a precise 1:1 model of the patient's skull and the mirrored contralateral head was made.

**FIGURE 2:**
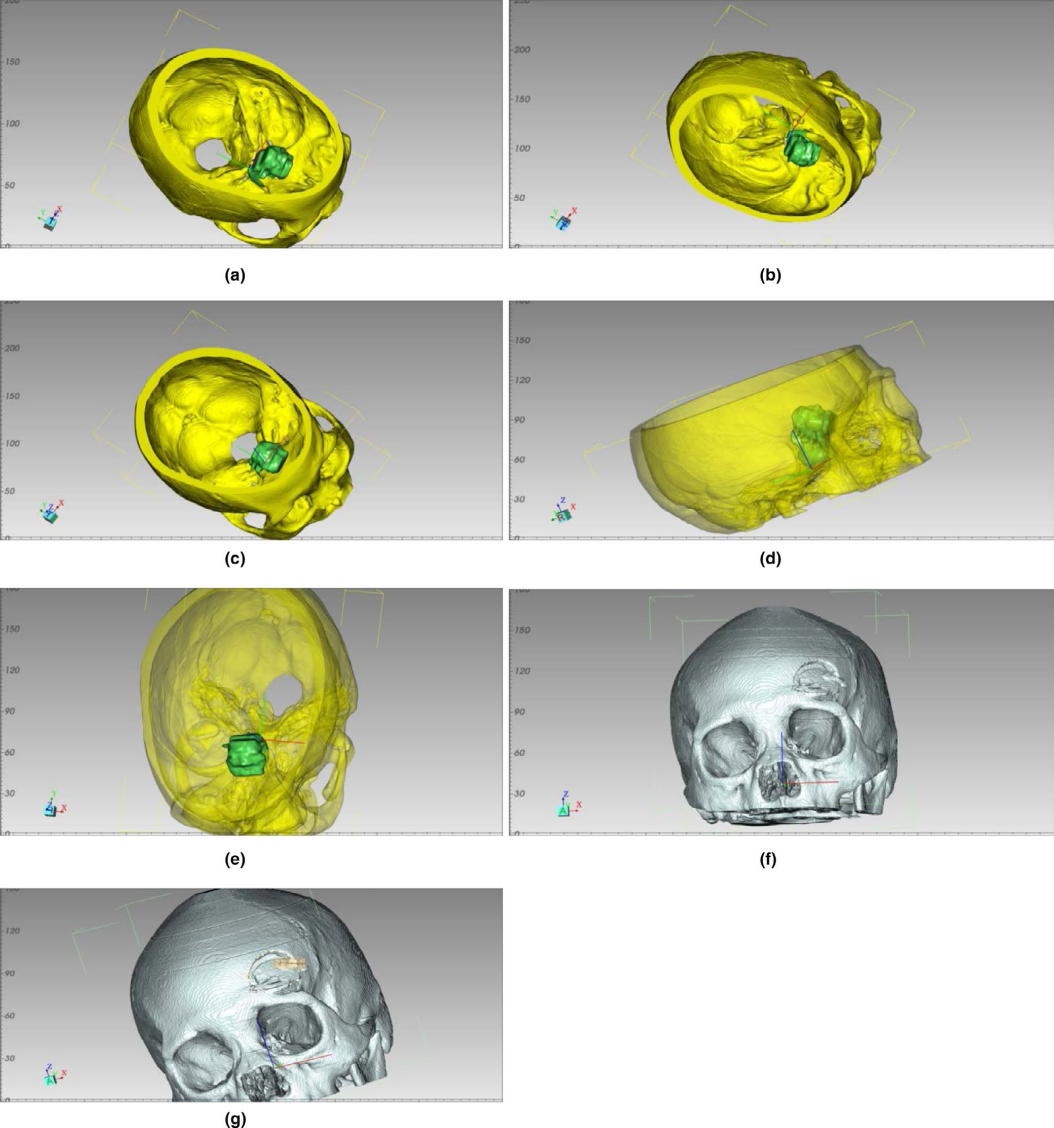
(a) Preoperative observation of the relationship between tumors and the skulls at 45 degrees above right (3D model); (b) The relationship between the tumors and the skull (3D model); (c) The relationship between skull and tumors (3D model) is observed at 45 degrees from the right anterior to the upper part of the skull; (d) Right fluoroscopy to observe the relationship between tumors and the skull (3D model); (e) Observing the relationship between tumors and the skull (3D model): The size of the bone window and surgical simulation after craniotomy is determined by observing in all directions, such as the ridge that can be encountered after craniotomy, the direction of surgical exploration and so on; (f) Postoperative outcomes in all directions can be observed and compared with preoperative planning (3D model); G. Through 3D software, the size and area of the window can be measured to provide assistance for precise surgery (3D model)

GOS score was used to evaluate the success and excellence of the operation in the two groups. The GOS scores of the two groups were evaluated according to the criteria. In the next six months, the subjects were followed up and investigated to record physical recovery and abnormalities, including sudden headache, subarachnoid hemorrhage, transient diabetes insipidus, fever, and so on (Figure 2).

### 2.5 Statistical analysis

Data were expressed as mean ± standard deviation. All statistical analyses were performed using PRISM version 4.0 (GraphPad Software, Inc., La Jolla, CA, USA). Differences between groups were analyzed using one‐way ANOVA and then Tukey multiple comparison tests were used as *post hoc* tests to compare group means. *p* < .05 was considered to represent a statistically significant difference.

## 3 RESULTS

### 3.1 General statistics of patients

This clinical trial enrolled 80 patients with intracranial aneurysms who were treated at our hospital from November 2017 to January 2019, including 42 males and 38 females with a mean age of 41.4 ± 6.4 years. Thirty cases had anterior communicating aneurysm, 22 had intracranial bifurcation aneurysm, 22 had internal carotid artery‐post‐tracheal aneurysm, and 6 had middle cerebral artery aneurysm. Most patients lacked a family history of genetic disease and had normal body movements, and no serious heart disease and mental illness. It was guaranteed to complete the experimental study. There were no significant differences in age, sex, and body weight between the two groups. The errors caused by the above reasons were eliminated to make the results more accurate (Table 1).

**TABLE 1:** Comparison of general data between the two groups of patients

Project	Conventional treatment group	3d printing assistant group	χ^2^/*t* value	*p* value
Age	41.17 ± 5.37	41.62 ± 6.57	2.351	.523
Gender (Male Female)	20:18	22:20	3.564	.146
BMI	22.67 ± 2.08	21.34 ± 2.19	4.266	.642
Brain genetic disease	2 (5.26%)	1 (2.38%)	3.335	.538

### 3.2 Patient Hunt‐Hess classification statistics

The patient underwent Hunt‐Hess classification during hospitalization, with 7 cases with grade 0, 56 cases with grade 1–3, and 17 cases with grade 4–5. There was no significant difference in Hunt‐Hess classification between the conventional treatment group and the 3D print aid group (*p* > .05) (Table 2).

**TABLE 2:** Hunt‐Hess classification at admission

Group	Level 0 (cases)	Level 1–3 (cases)	Level 4–5 (cases)
Conventional treatment group	3 (7.89%)	27 (71.05%)	8 (21.05%)
3D printing assisted group	4 (9.52%)	29 (69.05%)	9 (21.43%)
χ^2^ value	5.362	2.225	3.461
*p* value	0.254	0.165	0.377

### 3.3 Comparison of relevant indicators during 3D surgery

The progress of the operation of the two groups of patients during the experiment was recorded. Only the operative time was recorded except the anesthesia time. The operative time of the routine treatment group was significantly longer than that of the 3D printing assisted treatment group, including the time consumed by various emergencies and emergency plans encountered during the period. By comparing the incision area of patients, the incision area of the 3D print‐assisted treatment group was significantly lower than that of the conventional treatment group. Statistically, the incidence of surgical accidents was significantly lower in the 3D print‐assisted treatment group than in the conventional treatment group. There were significant differences between the two groups in the three surgical indicators (*p* < .05). It was indicated that the overall surgical progress was more reliable and operative than the conventional treatment group in the 3D print‐assisted treatment group (Table 3).

**TABLE 3:** Comparison of surgical conditions between the two groups

Group	Length of surgery (minutes)	Cutting area (mm^2^)	accidents
Conventional treatment group	120.35 ± 20.46	663.55 ± 13.54	6 (15.78%)
3d printing assistant group	70.13 ± 15.56	411.26 ± 10.38	1 (2.38%)
*t* value	6.625	4.06	6.196
*P* value	0.001	0.017	0.021

### 3.4 The 3D printing‐assisted treatment group had a lower rate of postoperative wound‐related complications

There were some complications in the two groups of patients during postoperative rehabilitation. Only one patient died within half a year after surgery. Among them, 12 cases had postoperative sudden headache, 3 had subarachnoid hemorrhage, 2 had transient diabetic injury, and 5 cases had fever. Twenty‐two patients had different degrees of postoperative complications. In statistical analysis of subarachnoid hemorrhage and transient urinary collapse, there was no similar situation in the 3D print assist group. In overall complications, the rate of complications in the 3D print‐assisted treatment group was significantly lower than that of the conventional treatment group (*p* < .05). The results showed that the rate of wound complications in the 3D print‐assisted treatment group was lower than that of the control group (Table S1).

### 3.5 3D printing assisted group had higher scores

**FIGURE 3:**
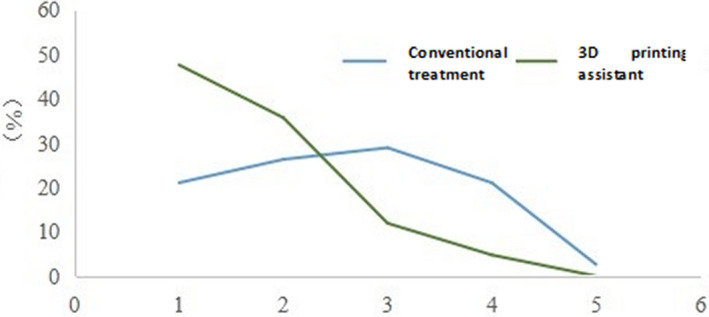
Postdischarge GOS scores in two groups of patients

All patients were followed up for six months, and the overall GOS score (postoperative rehabilitation, complications, side effects, and other discomforts) was recorded. In the 3D print‐assisted treatment group, the proportion of the 5th and 4th grades was higher. The proportion of the 3rd and 2nd grades in the conventional treatment group was higher, and the difference between the two groups was significant (*p* < .05) (Figure 3 and Table S2).

### 3.6 Patients with DSA review for aneurysms

DSA review was required 6–12 months after surgery. The patient's vascular image can be seen by digital silhouette technique. It was found that the residual rate of the conventional treatment group was significantly higher than that of the 3D print‐assisted group. The resection was incomplete. Two cases recurred in the conventional treatment group, and there was no recurrence in the 3D print‐assisted treatment group. The two groups had significant difference (*p* < .05) (Table S3).

## 4 DISCUSSION

In the late 1980s, the concept and indication of the keyhole method were proposed. Standard craniotomy provides sufficient space for examinations and instruments and provides space for different access angles to the target lesion (Bae, Lee, & Kim, 2010). At the same time, keyhole craniotomy provides the necessary space for microscopic observation. A wide variety of areas can be accessed by changing the microscopic angle of the view; however, only a fixed and limited approach angle toward a target is allowed through the keyhole (Zhuang, Cai, & Fu, 2017). It has been reported that the keyhole method is not limited, but rather a tailored adjustment method that minimizes brain exposure and retraction. Its advantages include minimal brain exposure, good cosmetic results, preservation of surrounding structures and short program time (Chen, Chen, & Huang, 2017). It also has some shortcomings: fewer opportunities to change plans, weaker microscopic illumination, and difficulty in controlling the proximal end of the paternal artery (Zhu, Mao, & Zhou, 2008). However, these shortcomings can be overcome by preoperative planning and imaging modes, intraoperative procedures and instruments such as endoscopes and specific equipment, as well as specific facilities. The keyhole approach includes squatting, underarm, under the pillow, between the hemispheres and across the cortex.

The 3D model is helpful for surgical diagnosis, improves consistency among observers and provides tactile feedback to allow preoperative planning of surgical approaches (including design and size reproduction for bone suture) on a virtual model (Cai, Ye, & Ling, 2019; Demartini, Matos, Dos Santos, & Cardoso‐Demartini, 2016). Multi‐planar reconstruction is a method for displaying 3D datasets and allows for the generation of cross‐sectional images, such as original two‐dimensional coronal, sagittal, and oblique images. The multi‐planar reconstruction of the curve is perpendicular to the cross‐sectional image of a particular curve made by the user (Ota, Matsukawa, & Noda, 2018; Zhang, Chen, Zheng, & Du, 2017). The 3D digital image has been upgraded to form a 3D material object. This provides important real‐world evidence for surgeons to make diagnoses and individual surgical plans, significantly improving the safety and effectiveness of surgery (Sughrue, Saloner, Rayz, & Lawton, 2011). With 3D printing technology, patient‐specific implant designs should have epoch‐making changes, and with the rapid development of 3D printing, the application of this technology is becoming more and more mature in orthopedics (He & Wan, 2018; Iosif & Biondi, 2019). In this study, we divided the patients into two groups. All the patients were operated through the eyebrow keyhole approach. The experimental group was assisted by 3D surface multiplanar imaging combined with 3D printing. The operative time, knife‐edge area, incidence of accidents, complications, and aneurysm eradication were recorded. Only from the GOS score, it can be seen that the 3D print‐assisted treatment group is better. Unfortunately, one patient in the conventional treatment group died in the third month after surgery, which was the only death in 80 subjects.

## 5 CONCLUSION

In summary, our results showed that 3D curved multi‐planar reconstruction images combined with 3D printing technology can achieve better surgical results and imaging results in keyhole microsurgery. Data Availability Statement: The datasets generated and analyzed during the current study are available from the corresponding author on reasonable request.

## CONFLICT OF INTEREST

The authors declare that they have no competing interests.

## AUTHORS' CONTRIBUTIONS

SL and YS contributed to the conception and design of the study. All authors participated in the clinical practice, including diagnosis, treatment, consultation, and follow‐up of patients. FW and WC contributed to the acquisition of data. FW and WC contributed to the analysis of data. SL wrote the manuscript. YS revised the manuscript. All authors approved the final version of the manuscript.

### Peer Review

The peer review history for this article is available at https://publons.com/publon/10.1002/brb3.1785.

## Supporting information

Table S1‐S3
