# Supplementary material for: Application of three dimensional (3D) curved multi‐planar reconstruction images in 3D printing mold assisted eyebrow arch keyhole microsurgery
Source: Brain Behav. 2020 Aug 14;10(10):e01785. doi: 10.1002/brb3.1785 (PMC7559599; doi:10.1002/brb3.1785)
Supplement: Supplementary file 1 — Table S1‐S3 [file BRB3-10-e01785-s001.docx]

**Supplementary Table 1 Comparison of postoperative wound-related complications in the two groups**

| Group | Sudden headache | Subarachnoid hemorrhage | Transient diabetes collapse | fever | total |
| --- | --- | --- | --- | --- | --- |
| Conventional treatment group | 9(23.68%) | 3(7.89%) | 2(5.26%) | 4(10.52%) | 18(47.36%) |
| 3D printing assistant group | 3(7.1%) | 0 | 0 | 1(2.38%) | 4(9.52%) |
| χ^2^ value | 4.325 | 3.761 | 6.196 | 4.163 | 3.755 |
| *P value* | 0.013 | 0.001 | 0.021 | 0.002 | 0.001 |

**Supplementary Table 2 GOS scores of the study patients**

| Group | 5 | 4 | 3 | 2 | 1 |
| --- | --- | --- | --- | --- | --- |
| Conventional treatment group | 8(21.05%) | 10(26.32%) | 11(28.95%) | 8(21.05%) | 1(2.63%) |
| 3D printing assistant group | 20(47.62%) | 15(35.71%) | 5(11.91%) | 2(4.76%) | 0 |
| χ^2^ value | 3.265 | 2.117 | 6.635 | 5.482 | 3.206 |
| *P value* | 0.002 | 0.016 | 0.001 | 0.024 | 0.013 |

**Supplementary Table 3 DAS review to detect aneurysm**

| Group | Aneurysm residue | Aneurysm recurrence |
| --- | --- | --- |
| Conventional treatment group | 4(10.52%) | 2(5.26%) |
| 3D printing assistant group | 1(2.38%) | 0 |
| χ^2^ value | 3.267 | 4.115 |
| *P value* | 0.024 | 0.001 |
